# Draft genome sequence of rhamnolipid-producing bacteria *Thermoanaerobacter thermocopriae* strain CM-CNRG TB177 isolated from an oil reservoir in Mexico

**DOI:** 10.1128/mra.00434-24

**Published:** 2024-08-20

**Authors:** Veronica Segovia, Pedro Martínez, Regina Hernández-Gama

**Affiliations:** 1 Unidad Profesional Interdisciplinaria de Ingeniería Campus Zacatecas, Instituto Politécnico Nacional, Zacatecas, Zacatecas, Mexico; 2 Centro de Investigación en Ciencia Aplicada y Tecnología Avanzada Campus Querétaro, Instituto Politécnico Nacional, Querétaro, Querétaro, Mexico; Portland State University, Portland, Oregon, USA

**Keywords:** *Thermoanaerobacter*, genomes, rhamnolipid, biosurfactants, oil reservoir

## Abstract

The draft genome of *Thermoanaerobacter thermocopriae* CM-CNRG TB177 isolated from an oil reservoir in Mexico was determined and annotated. The organism is a thermophilic and strict anaerobe bacterium that produces rhamnolipids, using glucose as a carbon source. The predicted genome size is 2,496,169 bp and 2,550 genes.

## ANNOUNCEMENT

The genus *Thermoanaerobacter* includes Gram-positive, thermophilic bacteria (1). Some species belonging to this genus have been studied for the production of biotechnologically important metabolites such as ethanol and enzymes, among others (2–4). The draft genome sequence presented here was particularly studied for rhamnolipid production (5). In addition to its biosurfactant production, the *Thermoanaerobacter thermocopriae* CM-CNRG TB177 strain is an extremophile bacterium, capable of thriving in temperatures ranging from 50°C to 80°C and in salt concentrations of up to 30 g/L of NaCl. These characteristics make this microorganism particularly valuable for industrial applications such as microbial enhanced oil recovery and mining.

The *T. thermocopriae* isolate was previously obtained from formation water at the Chicontepec oil field in Mexico (6). It was subsequently cultured in basal medium with glucose as the carbon source, as previously reported (7). Genomic DNA was extracted using the commercial ZymoBIOMICS DNA Miniprep Kit (Zymo Research, USA), and the DNA quality was verified using a NanoDrop One (Thermo Scientific, USA).

The DNA was sequenced twice. First, sequencing is performed using Illumina NextSeq 500 with the TruSeq DNA PCR-Free Sample Prep Kit in a 2 × 75 cycle configuration (Illumina, USA) by the Massive Sequencing Unit of the UNAM Institute of Biotechnology, constructing genomic DNA libraries with 350-bp fragments. The second sequencing was performed using Illumina MiSeq device in a 2 × 300 run format by the Genomic Services Laboratory of the National Laboratory of Genomics for Biodiversity (LANGEBIO), using the Illumina TruSeq DNA Nano Kit to construct the genomic DNA libraries using 550-bp fragments.

From the first sequencing, 28,471,116 paired raw sequences of 75 bp were obtained, and from the second sequencing, 1,341,482 paired raw sequences of 300 bp were obtained. The initial quality of the sequences was analyzed using the FastQC v0.11.9 program (8). Subsequently, the sequences were subjected to a cleaning process with the TrimmomaticPE v0.39 program (9). For the first sequencing data, the parameters used were a sliding window of 4:20, a head crop of one, trailing of two, and minimum length of 60. For the second sequencing data, the parameters used were a sliding window of 5:20 and the Ilumina clip to eliminate adaptors.

After cleaning 12,737,254 paired sequences of approximately 75 bp and 11,255,044 paired sequences of length 200–299 bp were obtained, with these reads, a coverage of 1,000 was estimated. From these sequences, genome hybrid assembly was performed using SPAdes version 3.15.3 (10), using only the assembly without sequence editing. The assembly generated 294 contigs, with a sequence length of 2,496,169, an *N*_50_ value of 94,187 bp; the longest contig was 252,232 bp, with a guanine and cytosine content of 34.68%. All tools were run with default parameters unless otherwise specified.

Genome annotation was performed using the Prokaryotic Genome Annotation Pipeline from the National Center for Biotechnology Information (11) version 6.7; as a result, 2,550 genes were obtained in the genome, including four complete rRNAs, 57 tRNAs, and four clustered regularly interspaced short palindromic repeats arrays. A genomic analysis was conducted using the GTDB-Tk toolkit (12), resulting in the assignment of *T. thermocopriae*.

## Data Availability

The sequence reads have been sumited to the Sequence Read Archive (SRA) under the accession number PRJNA1064994 (https://trace.ncbi.nlm.nih.gov/Traces/sra-reads-be/fastq?acc=SRR28816973 and https://trace.ncbi.nlm.nih.gov/Traces/sra-reads-be/fastq?acc=SRR28816972). This Whole Genome Shotgun project has been deposited at DDBJ/ENA/GenBank under the accession JBBLMQ000000000 and the Bioproject accesion number PRJNA1064994. The version described in this announcement is version JBBLMQ010000000.
